# Early Introduction of Sugar-Sweetened Beverages and Caries Trajectories from Age 12 to 48 Months

**DOI:** 10.1177/0022034520917398

**Published:** 2020-05-06

**Authors:** E. Bernabé, H. Ballantyne, C. Longbottom, N.B. Pitts

**Affiliations:** 1 Dental Public Health Group, Faculty of Dentistry, Oral & Craniofacial Sciences, King’s College London, London, UK; 2 National Health Service (NHS) Tayside, Dundee, UK; 3 Dental Innovation and Impact, Faculty of Dentistry, Oral & Craniofacial Sciences, King’s College London, London, UK

**Keywords:** dietary sugars, carbonated beverages, dental caries, infant, cohort studies, multilevel analysis

## Abstract

Early exposure to sweet tastes predicts similar food preferences and eating behavior in later life and is associated with childhood obesity. The aim of this study was to explore the associations of early (during the first year of life) and subsequent intake of sugar-sweetened beverages (SSBs) with 4-y caries trajectories among Scottish young children. We used data from 1,111 Scottish children who were followed annually from age 12 to 48 mo (4 sweeps in total). SSB intake was reported by parents in every sweep. SSB intake was broken down into 2 components, the initial SSB intake and the deviation over time from that initial value. Childhood dental caries was clinically determined (including noncavitated and cavitated lesions) every year. The association of SSB intake with baseline decayed, missing, and filled tooth surfaces (dmfs) (intercept) and rate of change in dmfs over time (slope) was examined in 2-level linear mixed-effects models, with repeated observations nested within children. Both the initial SSB intake and the deviation from the initial SSB intake were positively associated with steeper caries trajectories. By sweep 4, the predicted mean dmfs difference was 1.73 between children with low and high initial SSB intake (1 standard deviation below and above the mean) and 1.17 between children with low and high deviation from their initial SSB intake (1 SD below and above the mean). The findings of this prospective study among Scottish young children provide evidence that the introduction of SSBs during the first year of life can put children in a trajectory of high levels of dental caries. They support current recommendations to avoid sugars for very young children and interventions targeting early feeding practices for caries prevention.

## Introduction

The first year of life is a crucial stage for the development of eating behavior as infants transition from breast/formula-feeding to a greater variety of foods and beverages (Schwartz et al. 2011; Nicklaus 2017). The infant must learn not only how to eat but also what, when, and how much to eat (Nicklaus et al. 2019). Food preferences are shaped during this life period, and they track further on until adulthood (Appleton et al. 2018; Nicklaus et al. 2019). Therefore, an optimal beginning of complementary feeding (i.e., weaning) can help develop healthy dietary habits for life (Schwartz et al. 2011).

Children have an innate preference for sweet taste (Fidler Mis et al. 2017), and pleasure plays a central role in establishing food choices (Haines et al. 2019). There is evidence that early exposure to sweet tastes predicts similar food preferences and eating behavior in later life (Fidler Mis et al. 2017; Murray 2017). Children in both developed (Miles and Siega-Riz 2017) and developing countries (Pries et al. 2017) are exposed to sugar-sweetened beverages (SSBs) from the first year of life. This is worrisome given the high concentration of free sugars in these beverages and the aggressive marketing strategy of the soft drink industry. There is also evidence showing that such an early introduction of SSBs is associated with childhood obesity (Pan et al. 2014; Leermakers et al. 2015; Rose et al. 2017). These earlier findings are in line with the critical periods model, which emphasizes the timing of exposures, and the accumulation model, which emphasizes the duration of exposures, in life course epidemiology (Ben-Shlomo et al. 2016).

Only 2 previous longitudinal studies have evaluated the role of early introduction of SSBs in caries development (Thitasomakul et al. 2009; Chaffee et al. 2015). In one study, children who started having soft drinks at age 9 mo were at greater risk of developing dental caries by age 18 mo than their counterparts, after adjustment for confounders (Thitasomakul et al. 2009). In the other study, greater consumption of sugary food items, including SSBs, at ages 6 and 12 mo were associated with a greater incidence of severe dental caries at age 38 mo. However, only the intake of sugary food items at age 12 mo remained significantly associated after adjustment for confounders (Chaffee et al. 2015). What is missing in the literature is an assessment of the effects of initial and later consumption of SSBs on caries increment over time (trajectories). Furthermore, many studies use insensitive caries detection criteria and ignore precavitation lesions (Tinanoff et al. 2019). Both noncavitated and cavitated caries should be recorded to capture preventive and restorative needs (Pitts et al. 2019). This study explored the associations of early and subsequent consumption of SSBs with caries trajectories, from age 12 to 48 mo, in Scottish young children.

## Methods

This report follows the Strengthening the Reporting of Observational Studies (STROBE) guidelines. We used data from a population-based birth cohort in Scotland for which data were collected annually, including dental examinations (4 in total), from age 12 to 48 mo (Bernabe et al. 2017). Fluoride is not added to any drinking water supply in Scotland. The families of all 1,981 children born between April 1, 1993, and March 31, 1994, and residing in Dundee were invited to participate. They were approached by health visitors at the 8-mo child health review. Of them, 1,455 (73%) participated in sweep 1. There were 1,436, 1,292, and 1,412 participating children in sweeps 2, 3, and 4. The study was ethically approved by the Tayside Medical Research Ethics Committee.

Of the 1,419 young children with dental data on 1 or more sweeps, 308 (21.7%) were excluded because of missing values on SSB intake (*n* = 22) or relevant confounders (parental employment = 172, maternal age = 101, maternal smoking = 61, child birthweight = 37, toothbrushing frequency = 34, breastfeeding = 6, area deprivation = 1). The number of children with caries data were 1,099, 1,019, 871, and 957 in sweeps 1, 2, 3, and 4, respectively. Moreover, 781 (70.3%) had caries data in the 4 sweeps, 210 (18.9%) in 3 sweeps, 72 (6.5%) in 2 sweeps, and 48 (4.3%) in 1 sweep.

The outcome measure was early childhood caries indicated by the count of decayed, missing, and filled tooth surfaces (dmfs), including both noncavitated and cavitated lesions (Pitts et al. 2019; Tinanoff et al. 2019). The dmfs index was estimated for each sweep and treated as a repeated outcome measure. Parents were asked about the reason for dental extraction to confirm a tooth was missing due to caries. A single general dentist (H.B.) conducted all dental examinations, without assistance. She completed a 3-d training pack before every annual examination. The κ value for intraexaminer reliability from repeated examinations was 0.75 for all carious lesions (d_1–6_) and 0.67 for noncavitated lesions (d_1–2_). Neither professional cleaning nor toothbrushing was done before examinations. Children were examined in a supine position at baseline (lap-to-lap examination) and an upright position (child seated on a chair) at subsequent sweeps. Teeth were not cleaned or dried before examination. Caries was diagnosed at the caries-into-enamel threshold (d_1_), under artificial light and using direct vision only. Seventy-six percent of examinations were conducted within 3 mo of each child birthday.

The main exposure was the child SSB intake. SSBs were defined as any liquids containing added caloric sweeteners, such as soft drinks, fruit drinks, energy and sports drinks, and drinks sweetened after purchase (Miller et al. 2013). In every survey, parents reported how many times a day, on average, their children were given sugar-containing hot beverages and sugar-containing cold beverages. The child’s daily intake of SSBs was calculated as the sum of both responses and expressed as times per day. SSB intake was therefore a time-varying predictor (up to 4 data points per child).

Several maternal and child factors were selected for analysis as they could confound the hypothesized association (Mazarello Paes et al. 2015; Tinanoff et al. 2019). All confounders were measured at baseline (when children were 1 y old), apart from maternal education that was measured when children were 4 y old. Therefore, all confounders were treated as time invariant. Maternal predictors included age at delivery, smoking in pregnancy, education, parental employment, and level of deprivation of the area where the family lived. The latter was measured through the deprivation category (DEPCAT) score, which assigns every postcode sector in Scotland a deprivation score derived from the following census measures: overcrowding, male unemployment, car ownership, and the proportion of people in households in low social class. The DEPCAT score ranges from 1 for the most affluent to 7 for the most deprived postcode sectors. Participating families were grouped as affluent (DEPCAT score 1–2), intermediate (DEPCAT score 3–5), and deprived (DEPCAT score 6–7). Child predictors included sex, age (months), birthweight, breastfeeding, and toothbrushing frequency (Bernabe et al. 2017).

### Data Modeling

All analyses were performed in Stata (StataCorp). The impact of missing data on the results was assessed by comparing participants in the study sample with those excluded from the analysis, because of missing data, using the χ^2^ test. Thereafter, children’s SSB intake and dmfs values in every sweep were compared by each predictor. Student’s *t* test was used when comparing 2 groups, and Royston’s test for linear trends was used when comparing ordered groups.

The association between SSB intake and dmfs was examined in 2-level linear mixed-effects (LME) models, in which repeated observations (level 1) were nested within children (level 2) (Singer and Willett 2003; Twisk 2013). The time indicator in LME models was child age (continuous form, in months), which was centered at the average age in sweep 1 (12 mo). The coefficients (intercept and slope) for child age were estimated as random effects, allowing for individual variations in baseline dmfs (intercept) and the rate of change in dmfs over time (slope). The coefficients for other predictors were estimated as fixed effects. The time-varying SSB intake was decomposed into 2 indicators. The first was the child SSB intake at sweep 1 (labeled as initial SSB intake), which captured the between-child effects. The second was the increase or decrease in SSB intake, at each subsequent sweep, from that initial value (labeled as deviation from initial SSB intake), which captured the within-child effects (Singer and Willett 2003). Both indicators were treated as continuous.

In the LME model, 2 regression coefficients could be estimated for each predictor. The first captures differences in the intercept (baseline dmfs) between children with and without the predictor, whereas the second captures differences in the slope (growth trajectories in dmfs, hereinafter referred to as caries trajectories for simplicity) between children with and without the predictor (Singer and Willett 2003; Twisk 2013). In practice, these coefficients correspond to the predictor’s main effect and its interaction with time, respectively (Singer and Willett 2003). The conditional LME model included all associations with baseline dmfs (for all were theoretically relevant) and only the significant associations with caries trajectories (for model parsimony). To that end, the interaction of each predictor with child age was added one at a time to the simplest conditional LME model (one containing main effects for all predictors) for statistical testing. Interactions were added gradually, based on their contribution to model fit, until further addition did not improve model fit. The likelihood ratio test and the Akaike information criterion (AIC) were used to compare the fit of both (nested) models at every step. All variances and the covariance between intercept and slope of dmfs were estimated uniquely from the data (unstructured covariance matrix) (Singer and Willett 2003; Twisk 2013).

## Results

Data from 1,111 children were analyzed (baseline age: 12.8 ± 1.7 mo). There were differences between the study sample and those excluded because of missing data. Children in the study sample were more likely to be younger, to be breastfed, and to have had a lower caries experience; their mothers were older and more educated; and their families were more affluent (Appendix Table 1). The average daily SSB consumption was 1.8 ± 1.7 (range, 0 to 10), 4.3 ± 2.2 (range, 0 to 14), 4.3 ± 2.4 (range, 0 to 20), and 4.1 ± 2.2 (range, 0 to 20) times/d in sweeps 1, 2, 3, and 4, respectively. The correlation between initial SSB intake and deviation from initial SSB value at sweeps 1, 2, and 3 was −0.57, −0.51, and −0.59, respectively (all *P* < 0.001). In addition, the average dmfs per child was 0.1 ± 0.5 (range, 0 to 8; dmfs >0: 2.1%) in sweep 1, 0.7 ± 3.1 (range, 0 to 28; dmfs >0: 10.8%) in sweep 2, 1.5 ± 4.9 (range, 0 to 68; dmfs >0: 23.4%) in sweep 3, and 3.7 ± 7.9 (range, 0 to 62; dmfs >0: 45.8%) in sweep 4. The proportion of noncavitated carious lesions (d_1–2_) was 82.6%, 68.8%, 45.1%, and 34.6% in sweeps 1, 2, 3, and 4, respectively.

Breastfed children, those whose mother did not smoke in pregnancy, and those in affluent areas had significantly lower SSB consumption at every sweep (Table 1). Children with older, more educated mothers and employed parents also had lower intake of SSBs in 3 of the 4 sweeps. Sex differences in SSBs consumption were observed only at sweep 2, with greater SSB consumption among boys. On the other hand, children with employed parents, living in affluent areas, and with lower SSBs consumption had lower dmfs values at every sweep (Table 2). Breastfed children, those who brushed their teeth more often, and those with older, more educated and nonsmoking mothers also had significantly lower dmfs values in 3 of the 4 sweeps.

**Table 1.** Sample Description and SSBs Intake^a^ (Mean ± SD) in Every Sweep, by Confounders.

			Sweep 1 (*n* = 1,099)		Sweep 2 (*n* = 1,019)		Sweep 3 (*n* = 871)		Sweep 4 (*n* = 957)	
Baseline Predictors	*n*	%	Mean	(SD)	Mean	(SD)	Mean	(SD)	Mean	(SD)
Maternal age at birth										
16 to 24 y	334	30.1	2.2	(2.0)	4.7	(2.2)	4.8	(2.7)	4.4	(2.5)
25 to 34 y	696	62.7	1.7	(1.6)	4.1	(2.1)	4.1	(2.2)	4.0	(2.2)
35 to 44 y	81	7.3	1.3	(1.4)	4.2	(2.3)	4.6	(2.5)	3.8	(1.7)
* P* value for trend^b^			<0.001		0.004		0.067		0.040	
Maternal education										
No education	256	31.1	2.1	(1.8)	4.6	(2.2)	4.9	(2.4)	4.6	(2.2)
Secondary	296	35.9	1.7	(1.6)	4.3	(2.1)	4.6	(2.5)	4.2	(2.7)
A-levels	184	22.3	1.6	(1.6)	4.1	(2.1)	3.7	(2.5)	3.6	(1.5)
Degree or higher	88	10.7	1.8	(1.3)	3.3	(1.6)	3.4	(1.9)	3.6	(1.9)
*P* value for trend^b^			0.258		<0.001		<0.001		<0.001	
Maternal smoking in pregnancy										
Nonsmoker	740	66.6	1.7	(1.6)	4.0	(2.1)	4.0	(2.3)	3.7	(1.9)
Smoker	371	33.4	2.1	(2.0)	4.8	(2.2)	4.9	(2.5)	5.0	(2.7)
*P* value^b^			<0.001		<0.001		<0.001		<0.001	
Parental employment										
None employed	149	13.4	2.1	(2.1)	4.9	(2.5)	5.3	(2.9)	5.1	(2.4)
One employed	417	37.5	1.9	(1.8)	4.3	(2.3)	4.5	(2.5)	4.3	(2.3)
Both employed	545	49.1	1.8	(1.6)	4.1	(2.0)	4.0	(2.1)	3.8	(2.1)
*P* value for trend^b^			0.437		0.001		<0.001		<0.001	
Area deprivation										
Affluent	282	25.4	1.6	(1.4)	3.8	(2.0)	3.6	(2.3)	3.4	(1.8)
Intermediate	281	25.3	1.7	(1.7)	4.1	(2.1)	4.1	(2.0)	3.8	(1.8)
Deprived	548	49.3	2.1	(1.9)	4.6	(2.3)	4.8	(2.6)	4.6	(2.5)
*P* value for trend^b^			0.003		<0.001		<0.001		<0.001	
Child sex										
Boys	602	54.2	1.9	(1.8)	4.4	(2.1)	4.4	(2.4)	4.2	(2.2)
Girls	509	45.8	1.8	(1.6)	4.1	(2.2)	4.2	(2.4)	4.0	(2.3)
*P* value^b^			0.434		0.045		0.419		0.411	
Child birthweight										
≥2.5 kg	1048	94.3	1.8	(1.7)	4.3	(2.2)	4.3	(2.4)	4.1	(2.2)
<2.5 kg	63	5.7	1.8	(1.8)	3.8	(1.8)	4.2	(2.6)	4.2	(2.4)
*P* value^b^			0.720		0.102		0.697		0.857	
Child breastfeeding										
Never	598	53.8	2.1	(1.8)	4.4	(2.2)	4.5	(2.5)	4.3	(2.4)
<6 mo	318	28.6	1.6	(1.6)	4.2	(2.2)	4.1	(2.3)	3.9	(2.2)
≥6 mo	195	17.6	1.6	(1.6)	3.9	(2.0)	3.9	(2.2)	3.8	(1.8)
*P* value for trend^b^			0.001		0.004		0.003		0.037	
Child toothbrushing frequency										
No brushing	265	23.9	2.1	(1.9)	4.3	(2.4)	4.6	(2.6)	4.4	(2.6)
Once a day	352	31.7	1.6	(1.6)	4.2	(2.0)	4.2	(2.5)	4.1	(2.4)
Twice or more a day	494	44.5	1.9	(1.7)	4.3	(2.1)	4.2	(2.2)	4.0	(1.9)
*P* value for trend^b^			0.732		0.804		0.252		0.248	

a Child sugar-sweetened beverage (SSB) intake was obtained from parental reports on how many times a day, on average, their children were given sugar-containing hot and cold beverages. The child’s daily intake of SSBs was calculated as the sum of both responses and expressed as times per day.

b A *t* test was used when comparing nonordered groups and Royston’s test for linear trends when comparing ordered groups.

**Table 2.** Caries Experience (Mean Decayed, Missing, And Filled Tooth Surfaces ± SD) in Every Sweep, by Confounders.

	Sweep 1 (*n* = 1,099)		Sweep 2 (*n* = 1,019)		Sweep 3 (*n* = 871)		Sweep 4 (*n* = 957)	
Predictors	Mean	(SD)	Mean	(SD)	Mean	(SD)	Mean	(SD)
Maternal age at birth								
16 to 24 y	0.06	(0.45)	1.10	(3.89)	1.84	(6.10)	4.78	(8.50)
25 to 34 y	0.05	(0.49)	0.56	(2.74)	1.35	(4.56)	3.43	(7.77)
35 to 44 y	0.02	(0.22)	0.40	(2.05)	0.99	(2.70)	2.48	(5.51)
*P* value for trend^a^	0.281		<0.001		0.039		<0.001	
Maternal education								
No education	0.11	(0.76)	1.07	(4.02)	2.03	(4.84)	6.02	(9.72)
Secondary	0.07	(0.38)	0.59	(2.56)	1.61	(5.75)	3.98	(8.91)
A-levels	0.07	(0.64)	0.32	(1.26)	0.72	(2.74)	2.10	(5.00)
Higher	0.02	(0.18)	0.08	(0.65)	0.43	(1.80)	1.60	(3.37)
*P* value for trend^a^	0.117		<0.001		<0.001		<0.001	
Maternal smoking in pregnancy								
Nonsmoker	0.04	(0.38)	0.46	(2.43)	1.17	(4.79)	2.83	(6.58)
Smoker	0.09	(0.59)	1.20	(4.08)	2.04	(5.18)	5.54	(9.70)
*P* value^a^	0.088		<0.001		0.013		<0.001	
Parental employment								
None employed	0.11	(0.53)	1.70	(4.66)	3.16	(7.58)	7.16	(11.26)
One employed	0.06	(0.55)	0.66	(3.08)	1.54	(5.37)	3.83	(7.86)
Both employed	0.03	(0.35)	0.48	(2.49)	0.99	(3.51)	2.88	(6.62)
*P* value for trend^a^	0.001		<0.001		<0.001		<0.001	
Area deprivation								
Affluent	0.02	(0.36)	0.50	(2.90)	0.82	(3.56)	2.15	(6.69)
Intermediate	0.06	(0.40)	0.52	(2.10)	1.26	(4.11)	3.62	(8.42)
Deprived	0.07	(0.53)	0.90	(3.55)	1.92	(5.88)	4.65	(7.99)
*P* value for trend^a^	0.029		<0.001		<0.001		<0.001	
Child sex								
Boys	0.05	(0.43)	0.86	(3.62)	1.72	(5.54)	3.75	(7.76)
Girls	0.05	(0.49)	0.51	(2.25)	1.14	(4.12)	3.70	(7.97)
*P* value^a^	0.999		0.069		0.086		0.925	
Child birthweight								
≥2.5 kg	0.06	(0.47)	0.68	(3.00)	1.44	(4.97)	3.63	(7.68)
<2.5 kg	0.02	(0.13)	1.15	(4.15)	1.67	(4.41)	5.26	(10.17)
*P* value^a^	0.516		0.249		0.739		0.126	
Child breastfeeding								
Never	0.05	(0.38)	0.91	(3.60)	1.76	(5.61)	4.41	(8.27)
<6 mo	0.04	(0.49)	0.47	(2.33)	1.14	(4.46)	2.91	(7.09)
≥6 mo	0.09	(0.61)	0.47	(2.35)	1.05	(3.10)	2.97	(7.54)
*P* value for trend^a^	0.719		0.003		0.015		<0.001	
Child toothbrushing frequency								
No brushing	0.04	(0.50)	0.96	(3.26)	1.62	(3.82)	4.73	(8.63)
Once a day	0.05	(0.42)	0.77	(3.60)	1.62	(5.19)	4.44	(9.71)
Twice or more a day	0.07	(0.47)	0.52	(2.54)	1.26	(5.28)	2.71	(5.54)
*P* value for trend^a^	0.258		0.001		0.004		0.002	
Child SSB intake^b^								
Q1 (lowest)	0.02	(0.29)	0.43	(2.74)	1.38	(6.15)	2.08	(5.10)
Q2	0.04	(0.45)	0.54	(2.54)	1.11	(3.53)	4.25	(8.39)
Q3	0.09	(0.45)	0.98	(3.77)	1.92	(4.55)	4.38	(8.59)
Q4 (highest)	0.05	(0.38)	1.19	(3.50)	2.11	(7.23)	6.15	(11.02)
*P* value for trend^a^	0.041		<0.001		0.001		<0.001	

a A *t* test was used when comparing nonordered groups and Royston’s test for linear trends when comparing ordered groups.

b Child sugar-sweetened beverage (SSB) intake was categorized into quartiles for presentation purposes only.

The unconditional LME model showed that children’s dmfs increased by 0.09 units (95% confidence interval [CI], 0.08 to 0.10) per additional month in age. The unexplained variances for the intercept and slope were 0.21 (95% CI, 0.13 to 0.36) and 0.045 (95% CI, 0.036 to 0.053), respectively. The negative covariance between intercept and slope (–0.08; 95% CI, –0.11 to −0.06) suggested that children with the lowest baseline dmfs had the steepest caries trajectories. The conditional LME model is shown in Table 3. Maternal education, parental employment, area deprivation, maternal smoking during pregnancy, initial SSB intake, deviation from initial SSB intake, and child toothbrushing frequency were significantly associated with the rate of change in dmfs (slopes) when each was tested individually (Appendix Table 2). The final conditional LME model contained all individual predictors (reflecting their associations with baseline dmfs) and the interactions of maternal education, parental employment, maternal smoking during pregnancy, initial SSB intake, and deviation from initial SSB intake with child age (reflecting their associations with caries trajectories). The other 2 interactions did not improve the model fit further and were therefore dropped from the model (Appendix Table 2). The 5 predictors associated with the rate of change in dmfs are depicted in the Figure. To aid interpretation, initial SSB intake and deviation from initial SSB intake were plotted at their corresponding values for the mean, –1 SD (low), and +1 SD (high). By sweep 4, the predicted mean dmfs was 2.55 (95% CI, 1.74 to 3.36) for those with a low initial SSB intake (–1 SD = 0.2 times/d), 3.41 (2.87 to 3.96) for those with average initial SSB intake (mean = 2.2 times/d), and 4.28 (3.47 to 5.08) for those with high initial SSB intake (+1 SD = 4.2 times/d). Similarly, by sweep 4, the predicted mean dmfs was 2.90 (2.29 to 3.52) for those with a low deviation from initial SSB intake (–1 SD = −0.6 times/d), 3.50 (2.95 to 4.05) for those with average deviation from initial SSB intake (mean = 2.1), and 4.07 (3.41 to 4.73) for those with high deviation from initial SSB intake (+1 SD = 4.7). Finally, by wave 4, the predicted mean dmfs were 4.42 (3.28 to 5.55), 3.82 (2.83 to 4.81), 2.33 (1.09 to 3.57), and 2.07 (0.26 to 3.87) for mothers with no, secondary, A-levels, and higher education, respectively; they were 6.08 (4.49 to 7.68), 3.21 (2.31 to 4.11), and 2.82 (2.05 to 3.59) for children with none, 1, and both parents employed, respectively; and they were 4.39 (3.39 to 5.40) and 2.87 (2.20 to 3.55) for children whose mothers smoked and did not smoke during pregnancy, respectively. Residual diagnostics are presented in the Appendix Figure.

**Table 3.** Linear Mixed-Effects Model for the Association Between SSB Intake and Caries Trajectories among Scottish Young Children (*n* = 1,111).

	Coefficient^a^	[95% CI]	*P* Value
Fixed effects			
Maternal age at birth (reference: 16 to 24 y)			
25 to 34 y	−0.09	[–0.34, 0.17]	0.497
35 to 44 y	−0.06	[–0.51, 0.39]	0.797
Maternal education (reference: none)			
Secondary	−0.02	[–0.37, 0.33]	0.917
A-levels	0.16	[–0.23, 0.55]	0.427
Higher	0.14	[–0.37, 0.66]	0.581
Maternal smoking in pregnancy (reference: nonsmoker)			
Smoker	−0.13	[–0.41, 0.14]	0.352
Parental employment (reference: none employed)			
One employed	0.27	[–0.14, 0.68]	0.194
Both employed	0.28	[–0.14, 0.70]	0.195
Area deprivation (reference: affluent)			
Intermediate	−0.05	[–0.35, 0.24]	0.733
Deprived	0.06	[–0.22, 0.34]	0.680
Child age (centered at 12 mo)	0.14	[0.08, 0.19]	<0.001
Sex (reference: boys)			
Girls	−0.17	[–0.38, 0.04]	0.108
Child birthweight (reference: ≥2.5 kg)			
<2.5 kg	0.29	[–0.16, 0.75]	0.207
Child breastfeeding (reference: never)			
<6 mo	−0.05	[–0.30, 0.20]	0.705
≥6 mo	0.04	[–0.27, 0.35]	0.803
Child toothbrushing frequency (reference: no brushing)			
Once a day	−0.10	[–0.38, 0.19]	0.513
Twice or more a day	−0.09	[–0.36, 0.18]	0.500
Initial SSB intake	−0.10	[–0.17, –0.03]	0.006
Deviation from initial SSB intake	−0.14	[–0.22, –0.05]	0.001
Child age × maternal education (reference: no qualification)			
Secondary	−0.01	[–0.05, 0.03]	0.492
A-levels	−0.05	[–0.10, –0.01]	0.018
Degree or higher	−0.06	[–0.12, –0.003]	0.037
Child age × parental employment			
One employed	−0.07	[–0.12, –0.03]	0.002
Both employed	−0.08	[–0.13, –0.04]	<0.001
Child age × maternal smoking in pregnancy (reference: nonsmoker)			
Smoker	0.04	[0.01, 0.07]	0.016
Child age × initial SSB intake	0.01	[0.005, 0.02]	0.002
Child age × deviation from initial SSB intake	0.01	[0.005, 0.01]	<0.001
Intercept	−0.13	[–0.72, 0.45]	0.663
Random effects			
Variance (intercept)	0.13	[0.06, 0.25]	
Variance (slope)	0.040	[0.036, 0.045]	
Covariance (intercept, slope)	−0.07	[–0.10, –0.04]	
Residual variance	4.26	[4.01, 4.52]	

a A 2-level linear mixed-effects model, with repeated observations nested within children, was fitted. Regression coefficients are thus reported. The model-building process is shown in Appendix Table 2.

SSB, sugar-sweetened beverage.

**Figure.**
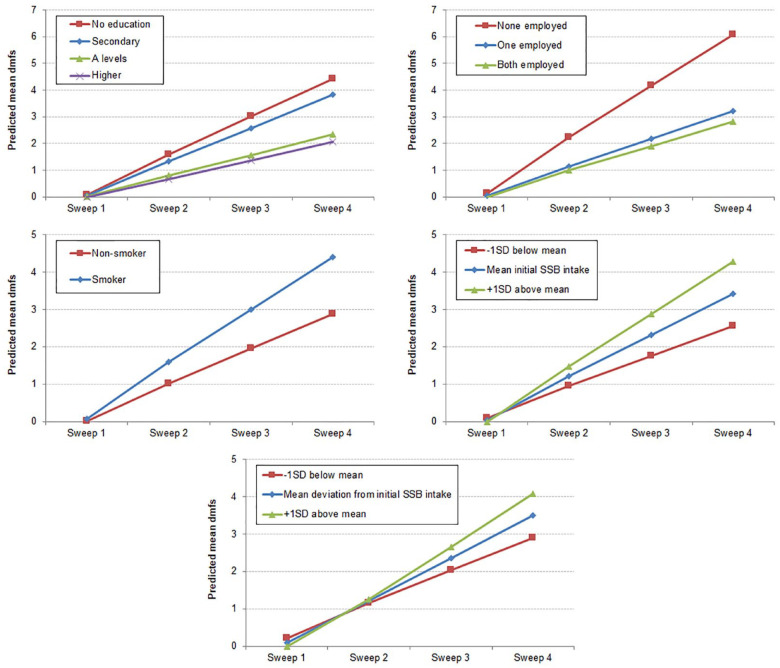
Predicted mean decayed, missing, and filled tooth surfaces (dmfs) according to maternal education, parental employment, maternal smoking in pregnancy, initial sugar-sweetened beverage (SSB) intake, and deviation from initial SSB intake. Predicted means were derived from the 2-level linear mixed-effects model in Table 3. For presentation purposes, low and high initial SSB intake values were calculated as 1 SD below and above the mean initial SSB intake (moderate), respectively. The same approach was used with deviation from initial SSB intake.

## Discussion

The findings of this study confirm our hypothesis that early introduction of SSBs, that is, by the end of the first year of life, is positively associated with caries trajectories from age 12 to 48 mo. This effect was independent of the intake of SSBs later in life. Our findings were robust to controls for established risk factors for early childhood caries.

Some strengths of this study were the large sample, multiple assessments for exposure and outcome, and recording the presence of noncavitated and cavitated caries (thus capturing both preventive and restorative needs). Some limitations must also be acknowledged. First, even though we used panel data to test our hypothesis, we cannot make any causal inferences. Second, we excluded around a fifth of participants from the analysis because they had missing data on 1 or more covariates. Participants in the study sample were, on average, healthier and wealthier than those excluded. Therefore, the present findings should not be extrapolated beyond the study sample. Third, children’s SSB intake was based on parental self-reports, which, although a common approach in dietary assessment, could introduce measurement bias. We could not identify the types of SSBs consumed or the amount of free sugars added to drinks. Although frequency and amount of consumption are moderately correlated (Bernabé et al. 2016), current recommendations on sugars intake emphasize limiting the amount, not the frequency, of consumption (World Health Organization 2015). In addition, information on other sources of sugars in children’s diet was not collected as part of the parental questionnaire, which meant that we could not adjust for them during modeling. However, the same analytical approach was used in previous longitudinal studies on SSB consumption and caries among children (Marshall et al. 2003; Park et al. 2015; Wigen and Wang 2015; Warren et al. 2016). Fifth, some confounders (marital status, parental employment, and maternal education) were treated as time invariant during the analysis because they were measured at a single sweep only. However, it is possible that these variables changed over time, which could have introduced residual confounding.

SSB intake was a time-varying exposure. Rather than representing it with a single measure in the analysis, we partitioned SSB intake into 2 constituent variables by centering it at a substantively meaningful value. We opted for using time 1 centering to decompose SSB intake into the time-invariant initial value and deviations from that starting point, thus capturing between-person and within-person variability, respectively (Singer and Willett 2003). Although alternative specifications could have been used to decompose within-child variability (such as grand-mean centering), our choice allowed us to identify the effects of early introduction and subsequent consumption of SSBs. This decision also gave us a clearer temporal sequence between exposure and outcome.

Our findings showed clear dose-response relationships with both measures of consumption, the initial SSB intake, and the deviation from that initial intake. As shown in the Figure, caries trajectories diverged more according to initial levels of SSB intake than to deviations from initial SSB intake. Taken together, the findings suggest that early introduction of SSBs places children on a trajectory of higher SSB consumption and greater caries increment. SSB intake during infancy is associated with continued consumption of these beverages in later life (Park et al. 2014) and poorer dietary quality among young children (Okubo et al. 2016). These studies are consistent with the development of sweet taste and preferences in the first year of life (Fidler Mis et al. 2017; Murray 2017).

It is worth mentioning that a negative association was found between breastfeeding and SSB intake. Children breastfed for up to 6 mo and those breastfed beyond 6 mo had, on average, lower intake of SSBs than those who were never breastfed at every sweep. Similar findings were reported earlier (Ha et al. 2017; Tovar et al. 2019). There is also evidence that breastfeeding duration shapes the development of food preferences and dietary intake later in childhood (Perrine et al. 2014; Beckerman et al. 2019). Formula milk contains sucrose and glucose, which are sweeter than lactose (i.e., the sugar in breastmilk) (Walker and Goran 2015). This area deserves further research.

The findings of this study have clear implications for policy and research. It is worrying that many infants are exposed to sweet tastes, such as SSBs, from such a young age. Our findings on SSBs along with previous findings on early introduction of sugars intake (Chaffee et al. 2015) build a body of evidence to support interventions targeting the first year of life to reduce the burden of dental caries among children. Indeed, the first 1,000 d of life (i.e., gestation to 2 y) are a fundamental stage for child development, where appropriate nutrition is paramount for brain and physical growth (Murray 2017; Nicklaus et al. 2019). What babies eat and drink is important for their health now and in the future (Nicklaus and Schwartz 2019). A number of international agencies recommend limiting the consumption of free sugars in general and SSBs in particular among infants and toddlers younger than 2 y (Fidler Mis et al. 2017; Vos et al. 2017; Haines et al. 2019; International Association for Paediatric Dentistry 2019). There is evidence that giving advice to mothers on healthy feeding practices during the first year of life can reduce childhood dental caries (Plutzer and Spencer 2008; Feldens et al. 2010). As for research, further longitudinal studies could look at other sources of free sugars during infancy as well as the potential interplay between breastfeeding, introduction of sugars, and caries increment.

## Conclusion

The findings of this prospective study among Scottish children provide evidence that the introduction of SSBs during the first year of life can put children in a trajectory of high levels of dental caries. Interventions targeting early feeding practices could help prevent dental caries. They could ensure parents are equipped with the necessary skills to give children the best start in life.

## Author Contributions

E. Bernabé, contributed to conception, design, data analysis, and interpretation, drafted and critically revised the manuscript; H. Ballantyne, C. Longbottom, contributed to data acquisition and interpretation, critically revised the manuscript; N.B. Pitts, contributed to data acquisition, analysis, and interpretation, critically revised the manuscript. All authors gave final approval and agree to be accountable for all aspects of the work.

## Supplemental Material

DS_10.1177_0022034520917398 – Supplemental material for Early Introduction of Sugar-Sweetened Beverages and Caries Trajectories from Age 12 to 48 Months Click here for additional data file. Supplemental material, DS_10.1177_0022034520917398 for Early Introduction of Sugar-Sweetened Beverages and Caries Trajectories from Age 12 to 48 Months by E. Bernabé, H. Ballantyne, C. Longbottom and N.B. Pitts in Journal of Dental Research
