# Supplementary material for: Early Introduction of Sugar-Sweetened Beverages and Caries Trajectories from Age 12 to 48 Months
Source: J Dent Res. 2020 May 6;99(8):898–906. doi: 10.1177/0022034520917398 (PMC7536523; doi:10.1177/0022034520917398)
Supplement: DS_10.1177_0022034520917398 – Supplemental material for Early Introduction of Sugar-Sweetened Beverages and Caries Trajectories from Age 12 to 48 Months [file DS_10.1177_0022034520917398.pdf]

**Appendix Table 1.** Comparison of baseline characteristics between children in the study sample and those excluded because of missing data.

| Baseline predictors | Lost to follow-up (n=308) | | Study sample (n=1111) | | p value |
|---|---|---|---|---|---|
| | **n** | **%** | **n** | **%** | |
| *Maternal age at birth* | | | | | <0.001 |
| 16-24 years | 108 | 49.8 | 334 | 30.1 | |
| 25-34 years | 97 | 44.7 | 696 | 62.7 | |
| 35-44 years | 12 | 5.5 | 81 | 7.3 | |
| *Maternal education* | | | | | 0.025 |
| None | 76 | 39.6 | 256 | 31.1 | |
| Secondary | 73 | 38.0 | 296 | 35.9 | |
| A-levels | 28 | 14.6 | 184 | 22.3 | |
| Higher education | 15 | 7.8 | 88 | 10.7 | |
| *Maternal smoking during pregnancy* | | | | | <0.001 |
| Non-smoker | 134 | 52.1 | 740 | 66.6 | |
| Smoker | 123 | 47.9 | 371 | 33.4 | |
| *Parental employment* | | | | | 0.051 |
| None employed | 23 | 15.8 | 149 | 13.4 | |
| One employed | 67 | 45.9 | 417 | 37.5 | |
| Both employed | 56 | 38.4 | 545 | 49.1 | |
| *Area deprivation* | | | | | <0.001 |
| Affluent | 41 | 13.4 | 282 | 25.4 | |
| Intermediate | 77 | 25.1 | 281 | 25.3 | |
| Deprived | 189 | 61.6 | 548 | 49.3 | |
| *Child's age at baseline, months* | 13.1 ± 2.0 | | 12.8 ± 1.7 | | 0.007 |
| *Child's sex* | | | | | 0.271 |
| Male | 156 | 50.6 | 602 | 54.2 | |
| Female | 152 | 49.4 | 509 | 45.8 | |
| *Child's birthweight* | | | | | 0.028 |
| ≥2.5kg | 255 | 90.8 | 1048 | 94.3 | |
| <2.5kg | 26 | 9.3 | 63 | 5.7 | |
| *Child's breastfeeding* | | | | | <0.001 |
| Never | 199 | 65.9 | 598 | 53.8 | |
| <6 months | 77 | 25.5 | 318 | 28.6 | |
| ≥6 months | 26 | 8.6 | 195 | 17.6 | |
| *Child's toothbrushing frequency* | | | | | 0.080 |
| No brushing | 86 | 30.3 | 265 | 23.9 | |
| Once a day | 80 | 28.2 | 352 | 31.7 | |
| Twice or more a day | 118 | 41.6 | 494 | 44.5 | |
| *Child's dmfs at baseline* | 0.18 ± 0.07 | | 0.05 ± 0.01 | | 0.005 |

**Appendix Table 2.** Model building selection process based on the Likelihood Ratio test and the Akaike Information Criterion (AIC).

| Models | Interaction with time | Likelihood ratio test | | | | AIC |
|---|---|---|---|---|---|---|
| | | Log-likelihood | df | $X^2$ value | p value | |
| M0 | Model with main effects | -8056.25 | 25 | | | 16162.49 |
| M1 | M0 + Age X Maternal age at birth | -8054.57 | 27 | 3.35 | 0.187 | 16163.14 |
| M2 | M0 + Age X Maternal education | -8045.03 | 29 | 22.44 | <0.001 | 16148.05 |
| M3 | M0 + Age X Maternal smoking during pregnancy | -8045.00 | 26 | 22.50 | <0.001 | 16141.99 |
| M4 | M0 + Age X Parental employment | -8043.25 | 27 | 26.00 | <0.001 | 16140.49 |
| M5 | M0 + Age X Area deprivation | -8050.37 | 27 | 11.75 | 0.003 | 16154.74 |
| M6 | M0 + Age X Child's sex | -8055.63 | 26 | 1.23 | 0.267 | 16163.26 |
| M7 | M0 + Age X Child's birth weight | -8056.21 | 26 | 0.08 | 0.780 | 16164.42 |
| M8 | M0 + Age X Child's breastfeeding | -8053.38 | 27 | 5.74 | 0.057 | 16160.76 |
| M9 | M0 + Age X Initial SSB intake | -8049.95 | 26 | 12.60 | <0.001 | 16151.89 |
| M10 | M0 + Age X Deviation in SSB intake | -8050.13 | 26 | 12.24 | <0.001 | 16152.25 |
| M11 | M0 + Age X Child's toothbrushing frequency | -8052.77 | 27 | 6.96 | 0.031 | 16159.53 |
| M12 | M4 + Age X Maternal education | -8035.38 | 31 | 15.73 | 0.003 | 16132.76 |
| M13 | M12 + Age X Area deprivation | -8034.79 | 33 | 1.17 | 0.557 | 16135.59 |
| M14 | M12 + Age X Maternal smoking during pregnancy | -8030.82 | 28 | 7.04 | 0.001 | 16117.64 |
| M15 | M14 + Age X child's sex | -8030.29 | 29 | 1.07 | 0.301 | 16118.57 |
| M16 | M14 + Age X child's birth weight | -8030.76 | 29 | 0.12 | 0.728 | 16119.52 |
| M17 | M14 + Age X child's breastfeeding | -8030.76 | 33 | 0.12 | 1.000 | 16127.52 |
| M18 | M14 + Age X Initial SSB intake | -8028.29 | 29 | 5.07 | 0.024 | 16114.57 |
| M19 | M18 + Age X Deviation SSB intake | -8020.41 | 34 | 15.75 | 0.008 | 16108.82 |
| M20 | M19 + Age X Child's toothbrushing frequency | -8017.07 | 36 | 5.43 | 0.056 | 16110.14 |

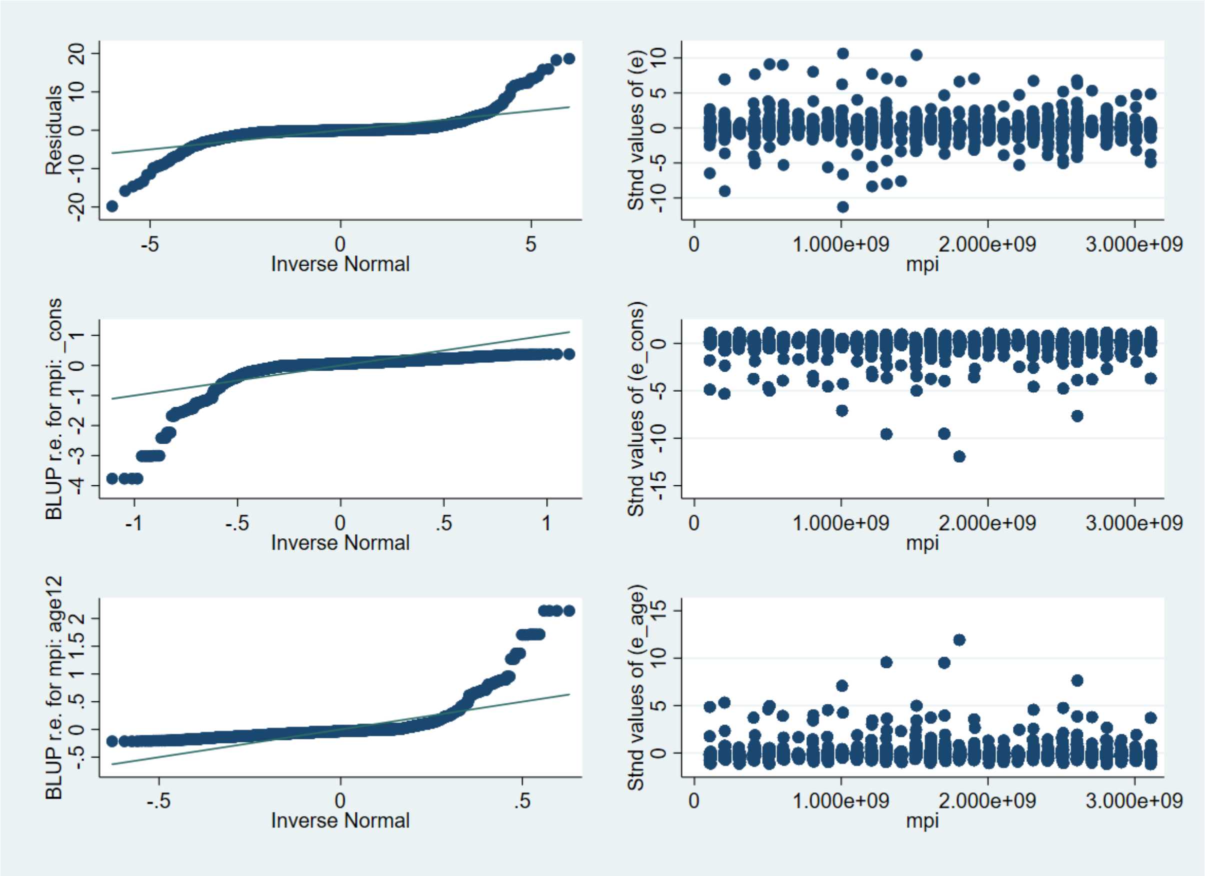


**Appendix Figure.** Examining the normality assumption of the final linear mixed effects (LME) model presented in Table 3. Left panel shows normal probability plots for the raw residuals at level-1 and level-2. Right panel shows plots of standardized residuals at level-1 and level-2 vs. *ID* numbers.
